# Loss of p21^Cip1/CDKN1A^ renders cancer cells susceptible to Polo-like kinase 1 inhibition

**DOI:** 10.18632/oncotarget.2844

**Published:** 2014-12-02

**Authors:** Nina-Naomi Kreis, Frank Louwen, Brigitte Zimmer, Juping Yuan

**Affiliations:** ^1^ Department of Gynecology and Obstetrics, J. W. Goethe-University, Frankfurt, Germany

**Keywords:** p21, Plk1 inhibitors, apoptosis, DNA damage, senescence

## Abstract

The deregulation of Polo-like kinase 1 is inversely linked to the prognosis of patients with diverse human tumors. Targeting Polo-like kinase 1 has been widely considered as one of the most promising strategies for molecular anticancer therapy. While the preclinical results are encouraging, the clinical outcomes are rather less inspiring by showing limited anticancer activity. It is thus of importance to identify molecules and mechanisms responsible for the sensitivity of Polo-like kinase 1 inhibition. We have recently shown that p21^Cip1/CDKN1A^ is involved in the regulation of mitosis and its loss prolongs the mitotic duration accompanied by defects in chromosome segregation and cytokinesis in various tumor cells. In the present study, we demonstrate that p21 affects the efficacy of Polo-like kinase 1 inhibitors, especially Poloxin, a specific inhibitor of the unique Polo-box domain. Intriguingly, upon treatment with Polo-like kinase 1 inhibitors, p21 is increased in the cytoplasm, associated with anti-apoptosis, DNA repair and cell survival. By contrast, deficiency of p21 renders tumor cells more susceptible to Polo-like kinase 1 inhibition by showing a pronounced mitotic arrest, DNA damage and apoptosis. Furthermore, long-term treatment with Plk1 inhibitors induced fiercely the senescent state of tumor cells with functional p21. We suggest that the p21 status may be a useful biomarker for predicting the efficacy of Plk1 inhibition.

## INTRODUCTION

The Cdk-interacting protein p21^Cip1/CDKN1A^ (p21) plays key roles in a broad range of cellular events like cell cycle regulation, apoptosis, differentiation, cytoskeletal dynamics, cell migration, gene transcription, DNA repair, reprogramming of induced pluripotent stem cells, aging and onset of senescence [1]. The transcription of p21 is regulated by p53-dependent [2] as well as -independent pathways [3]. Its expression is increased in response to various cellular stresses like DNA damage [4]. Interestingly, p21 functions not only as a tumor suppressor but also as an oncogene with a dual behaviour in different cellular processes [5,6], partially depending on its subcellular localization [1]. We have recently shown that p21 is important for a fine-tuned mitotic progression, its loss prolongs the duration of mitosis and results in severe mitotic defects in chromosome segregation and cytokinesis promoting genomic instability [7].

The Polo-like kinase (Plk) family is a group of highly conserved serine/threonine kinases. Five mammalian family members have been identified: Plk1, Plk2 (SNK), Plk3 (FNK or PRK), Plk4 (SAK) and Plk5 [8]. Plk1, the best studied member, is a key regulator of different cell cycle events and critical for multiple stages of mitosis including mitotic entry, spindle formation, chromosome segregation and cytokinesis [9]. Moreover, the overexpression of Plk1 in various tumor tissues is closely correlated with the poor prognosis of patients, and has been thus regarded as one of the most promising targets for molecular anticancer therapy [10,11]. The effect of Plk1 inhibition is well characterized, it induces mitotic arrest and apoptosis, leading further to a reduced proliferation *in vitro* and inhibited tumor growth *in vivo* [10]. The two functional domains of Plk1, the N-terminal kinase domain and C-terminal regulatory Polo-box domain (PBD) [10], offer multiple targeting strategies for developing specific small molecule compounds: (a) inhibitors targeting the ATP-binding pocket of the kinase domain, like BI 2536 [12,13] and BI 6727 (volasertib) [14,15], (b) inhibitors against the inactive conformation of the kinase domain, like SBE13 [16,17], and (c) inhibitors blocking the function of the unique PBD, like Poloxin [18]. In previous studies we have demonstrated that Poloxin, the first non-peptidic PBD inhibitor, specifically inhibits the Plk1-PBD, with a four-fold IC_50_ for the Plk2-PBD and an eleven-fold IC_50_ value for the Plk3-PBD *in vitro* [18]. Moreover, Poloxin targets Plk1 in a panel of cancer cell lines with a high specificity by showing prometaphase arrest, delocalization of Plk1 itself, reduction of γ-tubulin recruitment to centrosomes, defects in the mitotic spindle formation, activation of the spindle assembly checkpoint and induction of apoptosis, and it inhibits tumor growth *in vivo* [18-20].

Despite inspiring results of Plk1 inhibitors *in vitro*, the clinical data are less promising [11]. It is of importance to identify biomarkers, which contribute to the cytotoxicity of Plk1 inhibitors and help to select suitable cancer patients for this molecular intervention. Recently, we have reported that the cytotoxic response of various Plk1 inhibitors does not correlate with deficient p53, at least not in a direct manner, as functional p53 is required for an effective apoptosis induction upon Plk1 inhibition [21]. Since p21, the downstream effector of the p53 pathway, is involved in the regulation of proliferation, mitosis, apoptosis, stress response and survival, we wondered if the loss of functional p21 could affect the cytotoxicity of Plk1 inhibitors. In the present work, we have systematically addressed this issue.

## RESULTS

### HCT116 p21−/− cells respond more strongly to Plk1 inhibitors than HCT116 p21+/+ cells

**Figure 1:**
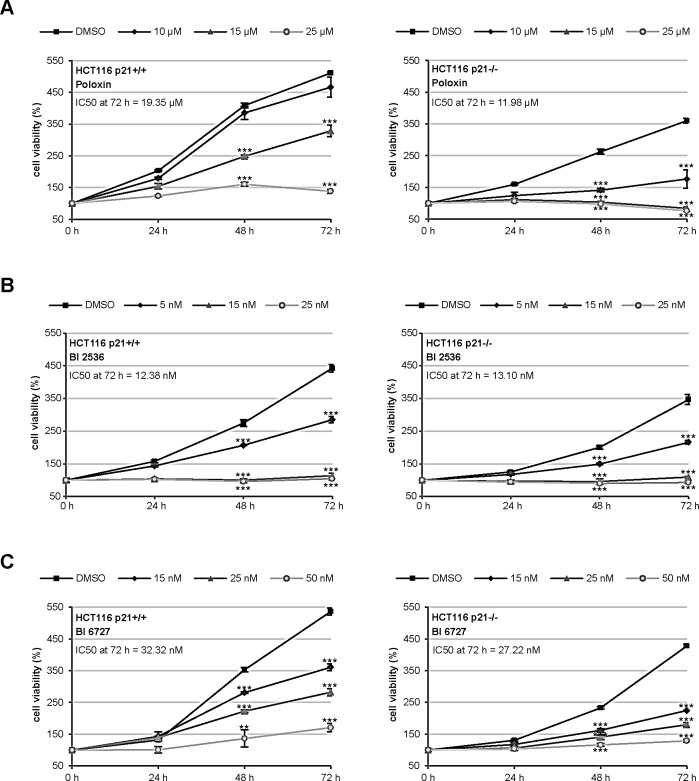
HCT116 p21−/− cells respond strongly to small molecule inhibitors against Plk1 (A-C) HCT116 cells were seeded in 96-well plates and treated with (A) 10, 15 or 25 μM Poloxin, (B) 5, 15 or 25 nM BI 2536 and (C) 15, 25 or 50 nM BI 6727 for 0, 24, 48 and 72 h. Cell viability was measured via CellTiter-Blue^®^ assay. DMSO treated cells served as vehicle control. The results are presented as mean ± SD and statistically analyzed compared to DMSO treated cells: **p < 0.01, ***p < 0.001. Right panels: HCT116 p21+/+ cells; left panels: HCT116 p21−/− cells. The IC_50_ values were calculated and indicated in the figure.

To address if the p21 status is a direct factor for the efficacy of Plk1 inhibitors, we have chosen the isogenic colon cancer cell lines HCT116 p21+/+ and HCT116 p21−/−, as they contain comparable cellular context except the p21 status and are very well characterized [22]. Using these cell lines, we tested the efficiency of the kinase domain inhibitors BI 2536 and BI 6727 [12-15] and the PBD inhibitor Poloxin [18-20]. While the BI inhibitors, like other inhibitors against a kinase domain, are highly potent, Poloxin, like other inhibitors targeting the protein binding domain, is specific yet less sensitive. HCT116 cells were treated with various concentrations of different Plk1 inhibitors for 24, 48 and 72 h, followed by cellular viability assays. HCT116 p21−/− cells expanded more slowly (Fig. 1A, B and C, right panel) than HCT116 p21+/+ cells (Fig. 1A, B and C, left panel), as previously described [7]. Interestingly, HCT116 p21−/− cells were obviously more sensitive to Poloxin by showing a strong inhibition of proliferation after the treatment with 10 μM Poloxin over 72 h and almost no proliferation upon 15 and 25 μM Poloxin (Fig. 1A, right panel), while HCT116 cells with p21 showed a moderate inhibition of proliferation even at 15 μM Poloxin (Fig. 1A, left panel). The IC_50_ calculation clearly accentuates the difference between these two cell lines upon Poloxin treatment for 72 h: the values were 19.35 μM for HCT116 p21+/+ and 11.98 μM for HCT116 p21−/− cells. Furthermore, both cell lines responded to BI 2536 and BI 6727 with a high sensitivity (Fig. 1B and C). A slightly enhanced proliferative inhibition was observed in HCT116 p21−/− cells treated with BI 6727 (Fig. 1C). The reduced viability in HCT116 p21−/− cells upon Plk1 inhibition was also observed by consecutive images of bright-field microscopy (Fig. S1).

### Mitotic arrest is more prominent in HCT116 p21−/− cells than in HCT116 p21+/+ cells

**Figure 2:**
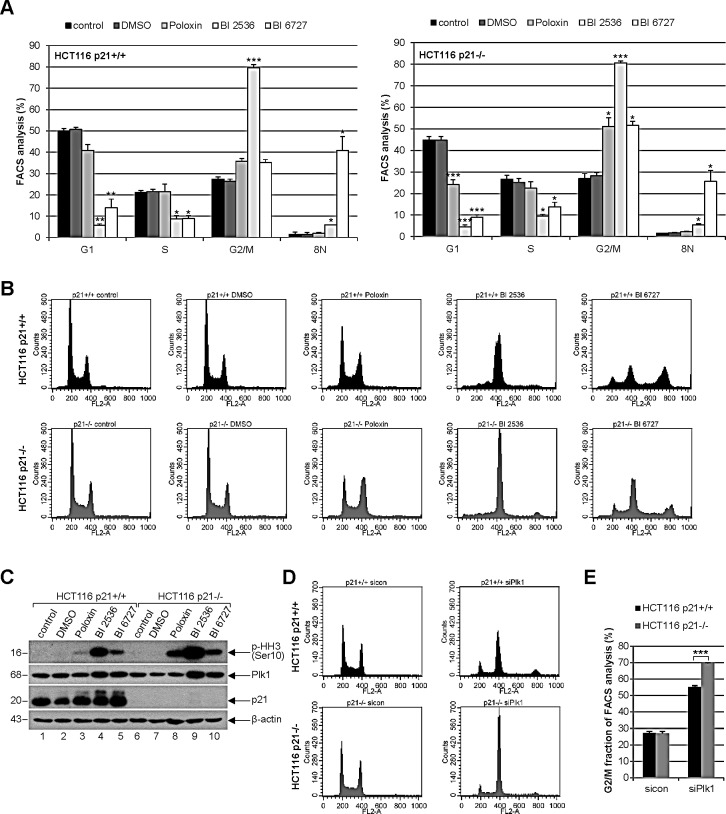
Strong mitotic arrest in HCT116 p21−/− cells upon Plk1 inhibition (A) Cells were treated with indicated Plk1 inhibitors (25 μM Poloxin, 25 nM BI 2536 or 25 nM BI 6727) for 24 h and cell cycle analyses were performed for HCT116 p21+/+ (left panel) and HCT116 p21−/− cells (right panel). The results are presented as mean ± SEM from three independent experiments and statistically analyzed compared to DMSO treated cells. *p < 0.05, **p < 0.01, ***p < 0.001. (B) Examples of FACS profiles are shown. Upper panel: HCT116 p21+/+ cells; lower panel: HCT116 p21−/− cells. (C) Cellular extracts were prepared for Western blot analyses with indicated antibodies. β-actin served as loading control. DMSO treated cells served as vehicle control. (D) HCT116 cells were treated with control siRNA or a mixture of two siRNAs against Plk1 (each 10 nM) for 24 h and cell cycle analyses were performed in triplicate. Examples of FACS profiles were shown. Upper panel: HCT116 p21+/+; lower panel: HCT116 p21−/−. (E) Results of (D) are presented as mean ± SD. ***p < 0.001. Control Western blot is shown in Fig. 4H.

To study the cell cycle distribution, both cell lines were treated with 25 μM Poloxin, 25 nM BI 2536 or 25 nM BI 6727 for 24 h and harvested for FACS measurements. Relative to HCT116 p21+/+ cells (Fig. 2A, left panel and Fig. 2B, upper panel), HCT116 p21−/− cells showed a more prominent G2/M arrest upon Poloxin and BI 6727 treatment (Fig. 2A, right panel and Fig. 2B, lower panel). By contrast, the G2/M peak was comparable in both cell lines upon BI 2536 treatment (Fig. 2A and B). In addition, as we recently observed [21], the treatment with BI 6727 produced an 8N peak, which was considerably higher in HCT116 cells with p21 than in cells deficient in p21 (Fig. 2A and B). Upon 24 h treatment with Plk1 inhibitors, the signals of the mitotic marker phospho-histone H3 (Ser10) were more intense in cells without p21 than in cells with it (Fig. 2C, 1^st^ panel), suggesting that Plk1 inhibition triggers more mitotic hindrance in cells lacking p21. To examine the specificity of Plk1 inhibition, we further treated cells with a mixture of two different siRNAs against Plk1 for 24 h and performed FACS analysis. It was apparent that Plk1 depletion triggered a more pronounced G2/M peak in HCT116 p21−/− cells compared to HCT116 p21+/+ cells (Fig. 2D). Further analysis showed that 79.6% of HCT116 p21−/− cells and 55.2% of HCT116 p21+/+ cells arrested at G2/M upon Plk1 depletion (Fig. 2E). The data are in line with our previous observations that p21 is required for a fine-tuned mitotic progression and cells deficient in p21 faced more troubles in mitosis, in particular, under stressful situations [7].

### Mitotic phenotype of HCT116 p21+/+ and HCT116 p21−/− cells upon Plk1 inhibitor treatment

**Figure 3:**
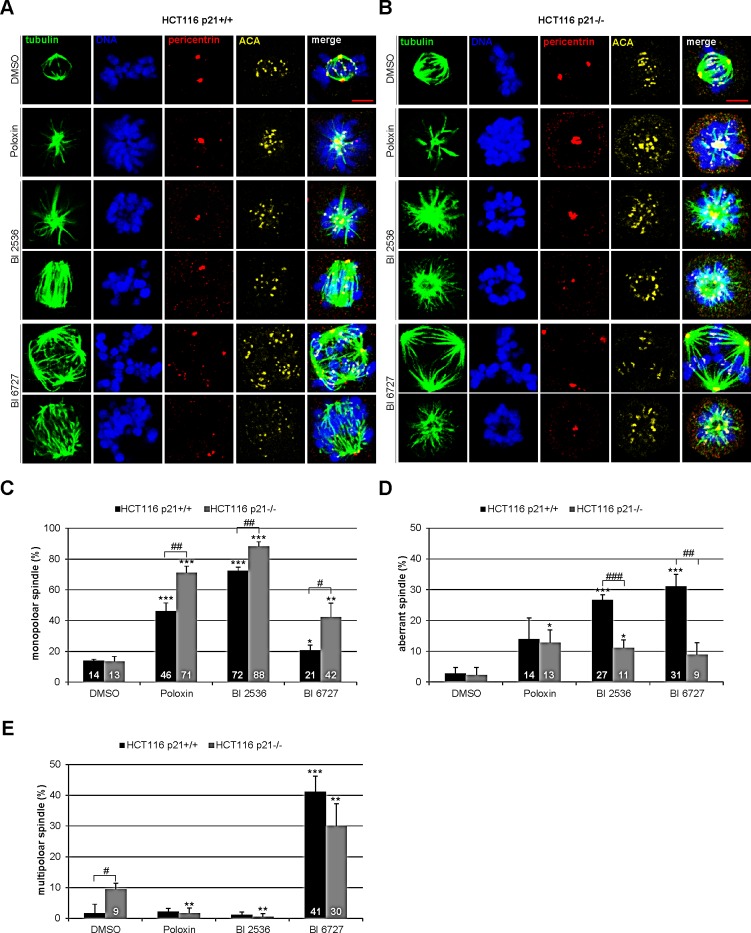
Mitotic phenotype of treated HCT116 cells HCT116 p21+/+ and HCT116 p21−/− cells were treated with 25 μM Poloxin, 25 nM BI 2536 or 25 nM BI 6727 for 24 h. Cells were stained with indicated antibodies and images were taken by confocal laser scanning microscopy. DMSO treated cells served as vehicle control. (A) Representatives from HCT116 p21+/+ cells. Scale bar: 5 μm. (B) Representatives from HCT116 p21−/− cells. Scale bar: 5 μm. (C-E) The spindle form of mitotic cells (n = 180) was evaluated and classified as (C) monopolar spindles, (D) aberrant spindles and (E) multipolar spindles. The results are presented as mean ± SD and statistically compared to DMSO treated cells (*p < 0.05, **p < 0.01, ***p < 0.001) or between HCT116 p21+/+ and HCT116 p21−/− cells (^#^p < 0.05, ^##^p < 0.01, ^###^p < 0.001).

Next we were interested in the mitotic phenotype induced by Plk1 inhibitors. Cells were treated for 24 h and stained for the microtubule marker tubulin, the centrosome marker pericentrin, the centromere/kinetochore marker ACA (anti-centrosome antibody) and DNA for microscopic evaluation. Compared to DMSO treated cells (Fig. 3A and B, 1^st^ panel), Poloxin and BI 2536 induced mainly monopolar spindles (Fig. 3A, 2^nd^ and 3^rd^ panel, Fig. 3B, 2^nd^ to 4^th^ panel), whereas BI 6727 produced primarily multipolar spindles (Fig. 3A and B, 5^th^ panel). Strikingly, all three inhibitors generated distinctively more monopolar spindles in HCT116 p21−/− cells relative to HCT116 p21+/+ cells (Fig. 3C), suggesting that loss of p21 strengths monopolar spindle formation induced by Plk1 inhibition. By contrast, the two BI inhibitors induced more aberrant spindles, defined as disorganized but bipolar spindles, in HCT116 p21+/+ cells (Fig. 3D). In addition, treatment with BI 6727 resulted in multipolar spindles which were more remarkable in HCT116 p21+/+ cells than in HCT116 p21−/− cells (Fig. 3E), supporting the 8N peak from the FACS analyses (Fig. 2A and B). The varied mitotic phenotypes could be ascribed to different working mechanisms of BI inhibitors and Poloxin. It could be also explained by varied remaining Plk1 activity after treatment with BI compounds or Poloxin, as described for selective inhibition of Plk1 by using chemical genetics [23]. In addition, differing from BI 2536, BI 6727 resulted in a substantial portion of multipolar cells, as previously reported [21,24]. We speculate that BI 6727 interferes with possibly not only Plk1 but also other kinases responsible for cytokinesis and centrosome duplication in treated cells.

### Poloxin induces strong apoptosis in cells deficient in p21

**Figure 4:**
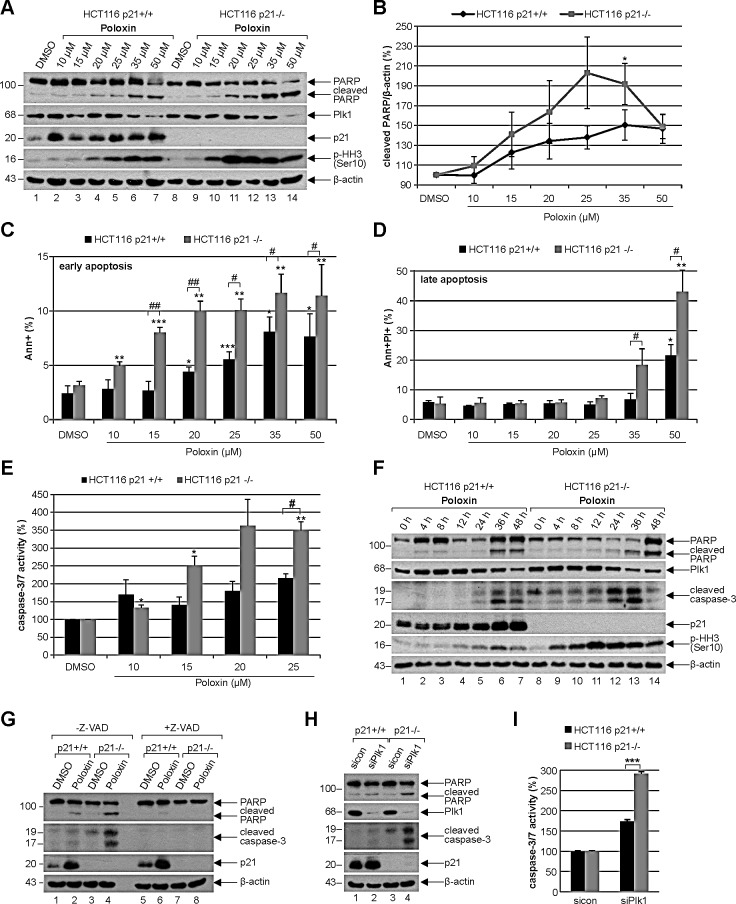
Poloxin induces strong apoptosis and more DNA damage in HCT116 cells without p21 (A) Western blot analysis of Poloxin concentration kinetics. HCT116 p21+/+ and HCT116 p21−/− cells were treated with 10, 15, 20, 25, 35 and 50 μM Poloxin for 24 h. Cellular lysates were prepared for Western blot analyses with indicated antibodies. DMSO treated cells served as vehicle and β-actin as loading control. (B) Quantification of the cleaved PARP signals from three independent Western blot analyses, relative to the corresponding β-actin signal, cells treated as in (A). The value of each DMSO control is defined as 100%. The quantification was performed with ImageJ (National Institutes of Health). The results are shown as mean ± SEM and statistically analyzed compared to its DMSO control (*p < 0.05). (C) Quantification of early apoptosis. Cells were treated as in (A). Annexin positive cells (Ann+) were evaluated by FACS using a Vybrant apoptosis kit. The results from four independent experiments are shown as mean ± SEM and statistically analyzed compared to DMSO control (*p < 0.05, **p < 0.01, ***p < 0.001) or between HCT116 p21+/+ and HCT116 p21−/− cells (^#^p < 0.05, ^##^p < 0.01). (D) Evaluation of late apoptosis. Cells were treated as in (A). Annexin and PI (propidium iodide) positive cells (Ann+PI+) were identified by FACS measurements (Vybrant). The results from four independent experiments are shown as mean ± SEM and statistically analyzed compared to DMSO control (*p < 0.05, **p < 0.01) or between HCT116 p21+/+ and HCT116 p21−/− cells (^#^p < 0.05). (E) HCT116 p21+/+ and HCT116 p21−/− cells were treated with DMSO, 10, 15, 20 and 25 μM Poloxin for 24 h. The relative activities of caspase-3/7 were measured. The results are based on three independent experiments, presented as mean ± SEM, and statistically analyzed compared to DMSO control (*p < 0.05, **p < 0.01) or between HCT116 p21+/+ and HCT116 p21−/− cells (^#^p < 0.05). (F) Western blot analysis of Poloxin time kinetics. HCT116 p21+/+ and HCT116 p21−/− cells were treated with 25 μM Poloxin for 0, 4, 8, 12, 24, 36 and 48 h. Western blot analyses were carried out with indicated antibodies. β-actin served as loading control. (G) HCT116 cells were treated without or with the pan-caspase inhibitor Z-VAD (10 μM) 1 h prior to Poloxin treatment (25 μM) for 24 h and Western blot analyses were performed with indicated antibodies and β-actin as loading control. (H) Cells were treated with control siRNA or a mixture of two siRNAs against Plk1 (each 10 nM) for 24 h and Western blot analyses were performed with indicated antibodies. β-actin served as loading control. (I) HCT116 p21+/+ and HCT116 p21−/− cells were treated as in (H). The relative activities of caspase-3/7 were measured in triplicate and presented as mean ± SD (***p < 0.001).

For further studies Poloxin was chosen, as it induced the strongest proliferation difference between HCT116 p21+/+ and HCT116 p21−/− (Fig. 1A). To analyze the effect of the specific PBD inhibitor Poloxin, cells were subjected to various concentrations for 24 h and harvested for Western blot analysis. Compared to HCT116 cells with functional p21, HCT116 p21−/− cells showed a stronger induction of apoptosis demonstrated by enhanced cleavage of poly (ADP-ribose) polymerase (PARP) upon treatment with Poloxin (Fig. 4A, 1^st^ panel), accompanied by a pronounced mitotic arrest evidenced by increased phospho-histone H3 (Ser10) (Fig. 4A, 4^th^ panel). As illustrated in Fig. 4B, the densitometric quantification of the cleaved PARP signals, based on three independent experiments, clearly displayed a stronger apoptosis induction in HCT116 p21−/− cells. The results were further underscored by measurements of early apoptotic cells (Fig. 4C, Ann+) as well as late apoptotic cells (Fig. 4D, Ann+PI+) via FACS and by evaluation of caspase-3/7 activity (Fig. 4E). Moreover, compared to HCT116 p21+/+ cells, an earlier and more intense apoptosis induction was underlined by Poloxin time kinetics showing an increased cleaved caspase-3 in HCT116 p21−/− cells (Fig. 4F, 3^rd^ panel). Again, a prominent mitotic arrest was demonstrated by enhanced phospho-histone H3 (Ser10) in HCT116 p21−/− cells (Fig. 4F, 5^th^ panel). To be sure that the caspase-dependent apoptosis pathway was mainly responsible for the cell death, cells were incubated with the pan-caspase inhibitor Z-VAD prior to Plk1 inhibitor treatment. The incubation with Z-VAD abolished the cleavage of PARP as well as caspase-3 in cells treated with Poloxin (Fig. 4G, 1^st^ and 2^nd^ panel), and both cleavage products were again more prominent in HCT116 p21−/− cells treated with Poloxin without Z-VAD (Fig. 4G, lane 4). Thus, cell death upon Plk1 inhibition depends mostly on the activation of caspases, leading to apoptosis induction. In addition, Plk1 depletion with a mixture of two different siRNAs induced stronger apoptosis in p21-deficient cells than in p21 wild type cells (Fig. 4H-I). The results suggest that HCT116 cells are more susceptible to Plk1 inhibition in the absence of p21 by triggering apoptosis.

### More apoptosis upon Poloxin treatment is also observed in U2OS and MDA-MB-231 cells depleted of p21

**Figure 5:**
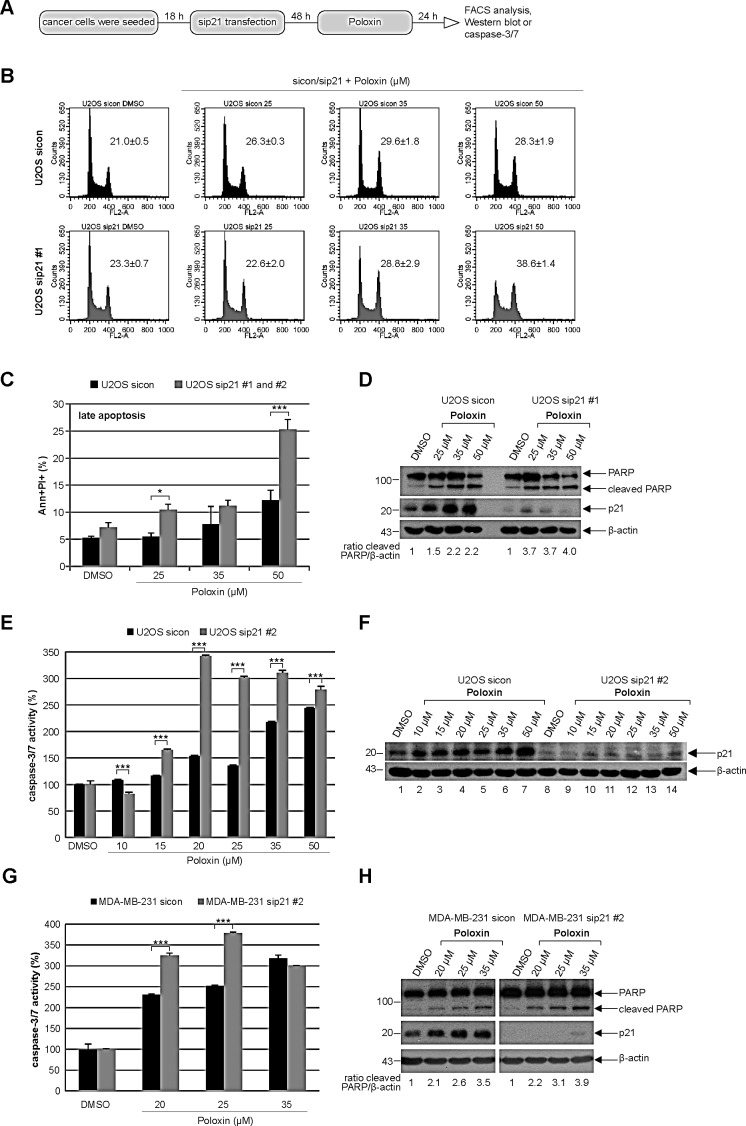
Tumor cells depleted of p21 with siRNAs show more apoptosis upon Poloxin treatment (A) Experiment schedule. (B) Cell cycle profiles of U2OS cells. Cells were treated with control siRNA (sicon, upper panel) or siRNA #1 targeting p21 (sip21 #1, lower panel) and then subjected to increasing concentrations of Poloxin. Cell cycle distribution was analyzed by FACS. The G2/M peak was quantified and results of the duplicate are shown as mean ± SD. DMSO treated cells served as vehicle control. (C) Treated cells were harvested for apoptosis measurements. Annexin and PI positive cells (Ann+PI+) were quantified and displayed as mean ± SEM from two independent experiments with two different siRNAs against p21. *p < 0.05, ***p < 0.001. DMSO treated cells served as vehicle control. (D) Western blot control of U2OS cells treated with sip21 #1. The cleaved PARP signal was quantified against β-actin by using ImageJ and displayed as ratio. β-actin served as loading control. (E) Evaluation of the relative activity of caspase-3/7. The results are shown as mean ± SD and statistical analyzed between the two groups (***p < 0.001). (F) Western blot control of U2OS cells treated with sip21 #2. β-actin served as loading control. (G) Metastatic breast cancer MDA-MB-231 cells were depleted of p21 and treated with indicated concentrations of Poloxin. The caspase-3/7 activity was measured and statistical analyzed between the two groups (***p < 0.001). (H) Western blot analyses with indicated antibodies. By using ImageJ, the cleaved PARP signal was quantified against β-actin and displayed as ratio. β-actin served as loading control.

To corroborate the data obtained by the usage of HCT116 cells, the osteosarcoma cell line U2OS was depleted of p21 and treated with Poloxin for 24 h (Fig. 5A). Relative to cells treated with control siRNA, 50 μM Poloxin triggered a stronger G2/M peak in U2OS cells depleted of p21 with siRNA #1 (Fig. 5B). Moreover, more apoptosis was induced by Poloxin treatment in U2OS cells treated with two different siRNAs against p21 (Fig. 5C), which was further supported by a stronger cleaved PARP signal in cells treated with siRNA #1 (Fig. 5D, 1^st^ panel and ratio) and an increased caspase-3/7 activity in cells treated with siRNA #2 (Fig. 5E). Furthermore, similar results were also obtained in the metastatic breast cancer cell line MDA-MB-231 depleted of endogenous p21 with siRNA #2 and treated with Poloxin for 24 h, which displayed an increase in caspase-3/7 activities (Fig. 5G) and in cleaved PARP compared to control cells (Fig. 5H, 1^st^ panel and ratio). Collectively, the data imply that the presence of functional p21 facilitates the survival of Plk1 inhibited cells by conferring resistance to mitotic arrest and apoptosis induction.

### Cells without p21 show more DNA damage upon Plk1 inhibition

**Figure 6:**
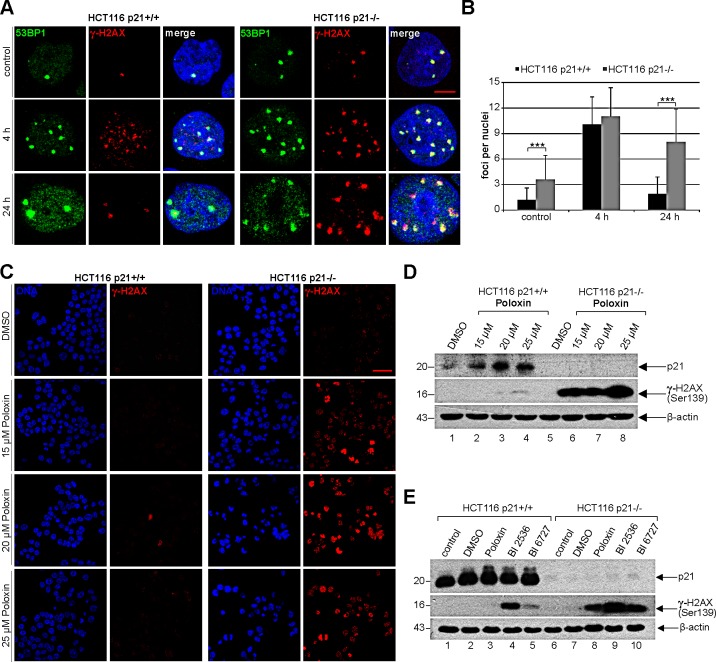
Cells without p21 show more DNA damage after Plk1 inhibition (A) HCT116 p21+/+ cells (left panel) and HCT116 p21−/− cells (right panel) were exposed to 2.0 Gy irradiation and stained for DNA damage markers 53BP1 and γ-H2AX (Ser139) at 4 h and 24 h after irradiation. Cells without irradiation served as negative control. Representative cells are shown. Scale bar: 5 μm. (B) Quantification of 53BP1 and γ-H2AX (Ser139) foci (n = 50, nuclei for each condition) and statistical analyzed between both cell lines (***p < 0.001). The results are presented as mean ± SD. (C) HCT116 p21+/+ cells (left panel) and HCT116 p21−/− cells (right panel) were treated with indicated concentrations of Poloxin for 24 h. Cells were stained for DNA and γ-H2AX (Ser139) and evaluated by confocal laser scanning microscopy. Scale bar: 50 μm. (D) Western blot analysis of HCT116 cells treated as in (C). β-actin served as loading control. (E) Western blot analysis of HCT116 cells treated with DMSO, 25 μM Poloxin, 25 nM BI 2536 or BI 6727 for 24 h. Cellular extracts were prepared for Western blot analyses with indicated antibodies. β-actin served as loading control.

Next we were wondered why cells with functional p21 were more capable of surviving the Plk1 inhibitor treatment. It has been reported that p21 is required for DNA repair and cells lacking p21 are more sensitive to DNA damage [25]. We hypothesized that the presence of p21 could impact the DNA damage response as well as repair contributing to the outcome of Plk1 inhibitor treatment. To test this, HCT116 p21+/+ and HCT116 p21−/− cells were irradiated, stained for DNA damage markers p53-binding protein 1 (53BP1) and γ-H2AX (Ser139), and the foci were evaluated at indicated time points. Indeed, untreated HCT116 p21−/− cells, compared to untreated HCT116 p21+/+ cells, had more foci indicative of double DNA strand breaks (Fig. 6A and B). 4 h after irradiation, regardless of the p21 status, the cells displayed higher numbers of 53BP1/γ-H2AX foci, which were almost disappeared in HCT116 p21+/+ cells 24 h after irradiation yet only slightly reduced in HCT116 p21−/− cells (Fig. 6A and B), suggesting that cells have indeed a defective DNA repair system in the absence of p21. Given that the balance between p21 expression and DNA damage is proposed to be crucial for DNA repair or apoptosis [4], we were then interested if the Poloxin treatment induced DNA damage and if it was affected by the p21 status in cells. To address this issue, HCT116 cells were treated with different Poloxin concentrations for 24 h and stained for γ-H2AX, the most sensitive and selective biomarker for DNA damage [26]. This kind of DNA damage has been known to precede caspase-3 activation and apoptosis in HeLa cells treated with siRNA against Plk1 [27]. Indeed, compared to cells with p21, HCT116 p21−/− cells displayed a stronger staining of γ-H2AX (Fig. 6C), further supported by Western blot analysis (Fig. 6D, 2^nd^ panel). Moreover, treatment with BI 2536 and BI 6727 showed also a remarkable increase in the γ-H2AX signals of cells deficient in p21 (Fig. 6E, 2^nd^ panel). Collectively, Plk1 inhibitors cause DNA damage indicated by the γ-H2AX staining, which cannot be repaired in HCT116 cells without p21 leading further to apoptosis.

### Cytoplasmic increase of p21 in HCT116 p21+/+ cells upon Plk1 inhibitor treatment

**Figure 7:**
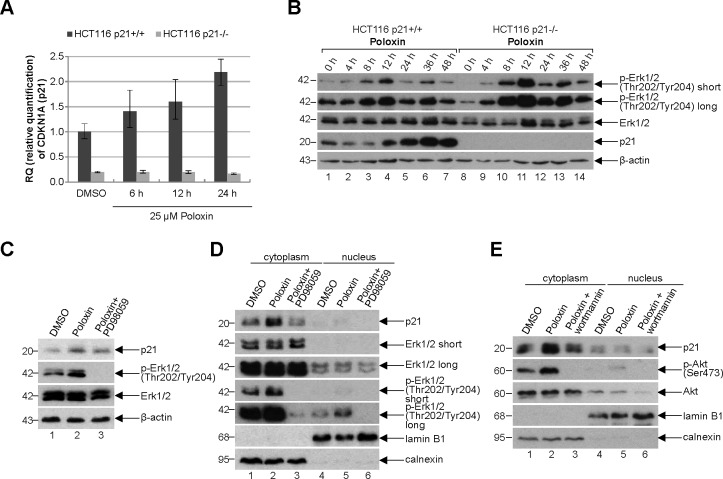
p21 is increased in the cytoplasm (A) Time-dependent increase of p21 mRNA upon Poloxin treatment. Relative amount of the p21 gene CDKN1A in HCT116 p21+/+ and HCT116 p21−/− cells after 25 μM Poloxin treatment for indicated time points. The results are presented by mean of RQ with minimum and maximum range. RQ: relative quantification of the fold by setting the DMSO value of HCT116 p21+/+ cells as 1. GAPDH served as endogenous control. (B) Western blot analysis of HCT116 p21+/+ and HCT116 p21−/− cells treated with 25 μM Poloxin for indicated time points. β-actin served as loading control. (C) Western blot analysis of DMSO, 25 μM Poloxin and Poloxin/PD98059 treated HCT116 p21+/+ cells. For the double treatment of Poloxin/PD98059, 50 μM PD98059 was provided to Poloxin treated cells for the last hour. β-actin served as loading control. (D) Western blot analysis. Cells were treated as in (C), fractionized into nucleus or cytoplasm, and cellular extracts were prepared. Calnexin served as cytoplasmic and lamin B1 as nuclear extract control. (E) Western blot analysis of DMSO, 25 μM Poloxin and Poloxin/wortmannin treated HCT116 p21+/+ cells. For the double treatment of Poloxin/wortmannin, 200 nM wortmannin was added to Poloxin treated cells for the last hour. Cells were fractionized into nucleus or cytoplasm and extracts were prepared. Calnexin served as cytoplasmic and lamin B1 as nuclear extract control.

To look deeply at possible mechanisms by which cells with functional p21 survive much better the Plk1 inhibition than cells without p21, HCT116 cells were treated with Poloxin and total RNA was isolated for mRNA evaluation using TaqMan^®^. The expression of p21 was increased upon Poloxin treatment in a time-dependent manner (Fig. 7A). Western blot analysis revealed that the protein level of p21 was also enhanced in HCT116 p21+/+ cells after Plk1 inhibition (Fig. 7B, 4^th^ panel). Intriguingly, the activating phosphorylation of Erk1/2 (extracellular-signal regulating kinase) at Thr202/Tyr204 was more prominent upon Poloxin treatment (Fig. 7B, 1^st^ and 2^nd^ panel). The increase of p21 induced by Poloxin in HCT116 cells (Fig. 7C, 1^st^ panel, lane 2) could be partially rescued by using PD98059, a selective inhibitor of the MAP (mitogen-activated protein) kinase cascade, proved by the abolished activating phosphorylation of Erk1/2 at Thr202/Tyr204 (Fig. 7C, 2^nd^ panel, lane 3). Western blot analysis with cytoplasmic and nuclear fractionized extracts revealed less p21 in the cytoplasm after treatment with PD98059 (Fig. 7D, 1^st^ panel, lane 3). The amount of p21 in the cytoplasm was also influenced by another kinase Akt/PKB (protein kinase B). Therefore, cells treated with wortmannin, a specific inhibitor of PI3K (phosphatidylinositide 3 kinase) of the Akt pathway, confirmed by the abolished phospho-Akt (Ser473) signal (Fig. 7E, 2^nd^ panel, lane 3), demonstrated less p21 in the cytoplasm (Fig. 7E, 1^st^ panel, lane 3). Thus, the results indicate that p21 is increased and probably stabilized in the cytoplasm upon Plk1 inhibition, which is regulated by the MAPK/Erk and PI3K/Akt pathways as well.

### Long-term treatment with Plk1 inhibitors induces senescence in HCT116 p21+/+ cells, whereas cells without p21 undergo apoptosis

**Figure 8:**
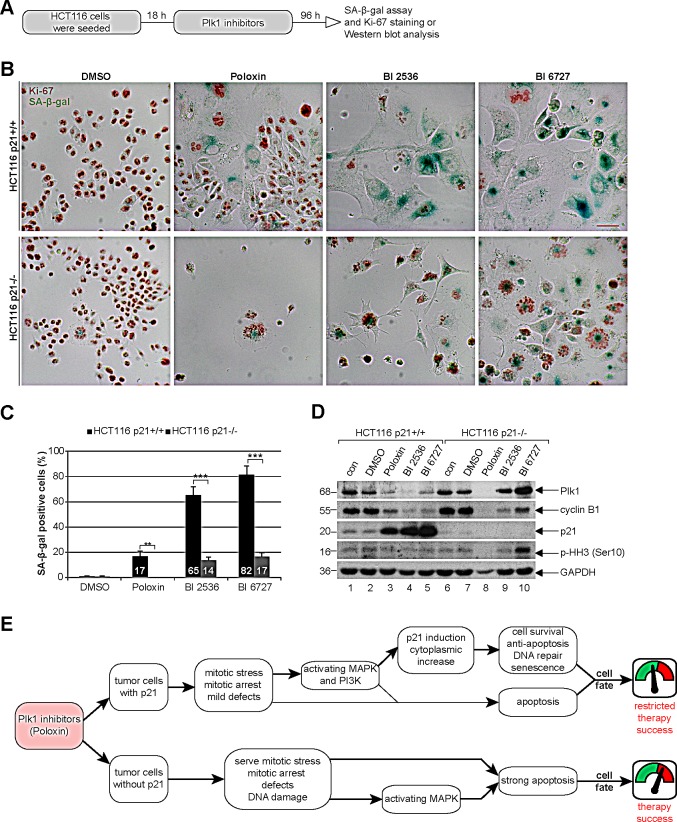
Poloxin induces senescence in HCT116 p21+/+, whereas cells without p21 undergo apoptosis (A) Experimental schedule. (B) HCT116 p21+/+ (upper panel) and HCT116 p21−/− cells (lower panel) were treated with DMSO, 25 μM Poloxin, 25 nM BI 2536 and 25 nM BI 6727 for 96 h. The cells were stained for SA-β-gal (blue-green) as well as the Ki-67 antigen (red-brown), a proliferation marker. Scale bar: 50 μm. (C) Evaluation of senescence-associated β-galactosidase (SA-β-gal) positive cells (at least n = 450 cells in each condition, except in Poloxin treated HCT116 p21−/− cells: n = 100). The results are presented as mean ± SD and statistically analyzed between both cell lines (**p < 0.01, ***p < 0.001). (D) Western blot analyses of long-term treated cells with indicated antibodies. GAPDH served as loading control. (E) Illustration of the impact of p21 on the efficacy of Plk1 inhibitors in cancer cells.

We wondered then the role of p21 in the long-term Plk1 inhibition. Cells were treated for four days with various Plk1 inhibitors for evaluation (Fig. 8A). In cells with p21, BI 6727 generated mostly enlarged multinucleated or multilobulated cells (Fig. 8B, upper panel, Fig. S1 and S2), whereas Poloxin caused massive cell death in the absence of p21 (Fig. 8B, lower panel, and Fig. S1). Strikingly, cells with functional p21 underwent substantially more senescence after the long-term treatment with various Plk1 inhibitors (Fig. 8B), demonstrated by using a SA-β-gal (senescence-associated β-galactosidase) assay, a well-established assay for the detection of senescent cells [28], in combination with the co-staining of the proliferation marker Ki-67 to avoid pseudo-positivity (Fig. 8B). Further analysis revealed that BI 2536 and BI 6727 generated 4.6 and 4.8 fold more senescent cells in HCT116 p21+/+ cells, respectively, compared to HCT116 p21−/− cells (Fig. 8C). In addition, the proliferation markers Plk1 and cyclin B1 were hardly detectable by Western blot analysis in HCT116 p21+/+ cells after the long-term inhibition (Fig. 8D, 1^st^ and 2^nd^ panel, lanes 3 to 5), suggestive of cells being non-proliferative but senescent. Proliferation markers were unobservable in HCT116 p21−/− cells treated with Poloxin, due to massive apoptosis induction indicated by reduced GAPDH (Fig. 8D, lane 8).

## DISCUSSION

In the present work, we show that p21 impacts the efficacy of Plk1 inhibition in tumor cells. Compared to HCT116 p21+/+ cells, HCT116 cells without p21 are more vulnerable to Plk1 inhibition by showing a stronger induction of mitotic arrest (Fig. 2), DNA damage (Fig. 6), apoptosis (Fig. 4) and inhibition of proliferation (Fig. 1). These results were further corroborated by using different human cancer cell lines depleted of p21 (Fig. 5), indicative of p21 being a general protector against Plk1 inhibition. Furthermore, in the presence of p21, long-term treatment of Plk1 inhibitors resulted in more senescence in HCT116 cells (Fig. 8A to D). Our data suggest that the Cdk-interacting protein p21 is a useful biomarker for predicting the efficacy of Plk1 inhibition in tumor cells.

For an effective anticancer therapy, it is desirable to ensure that the damage induced by small molecule compounds like Plk1 inhibitors is strong enough to drive tumor cells undergoing apoptosis otherwise a drug resistance phenotype will be provoked. In this regard, it is unfortunately known that p21 favors the activation of the anti-apoptotic pathways and the implementation of senescence, whereas the lack of p21 facilitates apoptosis [29-31], supporting the role of p21 as a threshold modulator of apoptosis as proposed [4,32-34]. Obviously, the presence of p21 protects tumor cells from severe damage upon Plk1 inhibition. Consequently, non-severe DNA damage induces the expression of p21, which causes a cell cycle arrest [35,36] and favors an efficient DNA repair [25,37,38], resulting in a resumed cell cycle. By contrast, the inactivation of p21 is important for switching from cell cycle arrest to apoptosis [32,39]. In line with these notions, our data indicate that the presence of functional p21 facilitates the survival of tumor cells upon Plk1 inhibitor treatment by conferring resistance to mitotic arrest and protection against apoptosis. Since p21 is required for DNA damage repair [25,37,38] and Plk1 is necessary for the recovery of DNA damage [40], Plk1 inhibition in p21-deficient cells could therefore synergize the cytotoxicity of Plk1 inhibitors and strength the induction of apoptosis, which is possibly dependent on p53, as functional p53 is required for a strong induction of apoptosis [21].

Furthermore, we demonstrate that Plk1 inhibition induces mitotic stress by defective spindle formation (Fig. 3), which increases the mRNA and protein level of p21 in a time-dependent manner (Fig. 7A and B). Interestingly, the increased p21 is localized to the cytoplasm (Fig. 7D and E), associated with the activation of the MAPK/Erk and PI3K/Akt pathways (Fig. 7B to E). It is well known that p21 can act as a tumor suppressor as well as an oncogene, the later correlates tightly with its cytoplasmic localization [1], frequently observed in human tumors [41]. Cytoplasmic p21 causes the suppression of apoptosis induced by DNA damage [29,42]. Its anti-apoptotic properties are mediated through the inhibition of various caspases and apoptotic effectors [41]. Importantly, p21 phosphorylations by Erk2 or Akt/PKB result in its cytoplasmic translocation and stabilization [1]. We show here that Plk1 inhibition activates the MAPK/Erk and PI3K/Akt pathways (Fig. 7B to E), likely responsible for increased p21 in the cytoplasm of HCT116 cells (Fig. 7D and E), leading further to the inhibition of apoptosis and survival of treated tumor cells. The importance of our results is underscored by other studies showing that increased cytoplasmic p21 due to Akt phosphorylation contributes to cancer progression, therapy resistance and poor prognosis [34,43-46]. In fact, a recent study has highlighted the significance of cytoplasmic p21 *in vivo* demonstrating an accelerated tumor onset and lung metastasis by generating transgenic mice expressing its Akt-phosphorylated active form (p21T145D) in the mammary epithelium [47].

Plk1 inhibitors are currently undergoing various clinical trials [48], it is thus important to study its response in tumor cells after a long-term treatment. Interestingly, a distinctive induction of senescence in p21 wild type cells was observed upon four days treatment, especially with BI 2536 or BI 6727, characteristic of being flattened, enlarged, multinucleated, SA-β-gal-positive and Ki-67-negative (Fig. 8 A to D, Fig. S1 and S2), whereas a strong apoptosis was induced in cells lacking p21 (Fig. 8A to D, Fig. S1). These results are supported by a previous study showing that p21 was responsible for senescence induction in cells treated with low concentrations of camptothecin whereas HCT116 cells without p21 underwent apoptosis [31]. Our data are further underlined by developmental studies, in which apoptosis but not senescence was observed in cells without p21 [49,50]. Importantly, it has been reported that partial inhibition of the activity of Plk1 by using chemical genetics or its depletion with siRNA induces cellular senescence [23,51]. Together these data indicate that Plk1 inhibition in p21-deficient cells favors the induction of senescence. Given the supportive role of senescent cells for tumor cell development, via a profound secretory phenotype with pro-inflammatory characteristics [52] contributing to therapy resistance [53], it should be kept in mind that tumor cells which survived Plk1 inhibitor treatment could contribute to a more aggressive cancer development.

In summary, p21 is crucial to determine the fate of tumor cells treated with Plk1 inhibitors, in particular Poloxin (Fig. 8E). In the presence of p21, Plk1 inhibition, along with an induction of mitotic arrest, enhances strikingly the expression of p21 and activates MAPK/Erk and PI3K/Akt pathways, which likely stabilizes p21 in the cytoplasm of treated tumor cells. Increased cytoplasmic p21 facilitates DNA damage repair, confers resistance to apoptosis and favors senescence induction in tumor cells, leading to cell survival and a limited therapy success accompanied by a small fraction of cells undergoing apoptosis (Fig. 8E). In contrast, cells without p21 displayed a pronounced mitotic arrest, irreversible DNA damage, the activation of apoptosis favorable MAPK/Erk pathway [54] and intense apoptosis induction (Fig. 8E), strongly indicative of a high efficacy of Plk1 inhibitors in p21-deficient tumor cells.

## METHODS

### Cell culture, inhibitors, siRNA transfections and irradiation

HCT116 p21+/+, HCT116 p21−/−, U2OS and MDA-MB-231 cells were cultured as instructed. To compensate the faster proliferation HCT116 p21+/+ cells were seeded 10% less than HCT116 p21−/− (except: proliferation assays). BI 2536 and BI 6727 were purchased from Selleck Chemicals LLC (Houston, USA). The pan-caspase inhibitor Z-VAD-FMK (Z-VAD) was obtained from Enzo Life Science GmbH (Lörrach), DMSO from Sigma-Aldrich (Taufkirchen), PD98059 from Merck Millipore (Darmstadt) and wortmannin from Cell Signaling (Beverly, USA). siRNA (20 nM) transient transfections were performed as previously described [7]. Regarding Plk1 depletion, two different siRNAs (each 10 nM) were mixed to avoid off-target effects. siRNAs targeting p21 (sense: ACACCUCCUCAUGUACAUAUU and antisense: AAUAUGUACAUGAGGAGGUGU; designated as sip21 #2) or Plk1 (sense 1: GAUCACCCUCCUUAAAUAU and antisense 1: AUAUUUAAGGAGGGUGAUC, sense 2: GAAGAAUGAAUACAGUAUU and antisense 2: AAUACUGUAUUCAUUCUUC) were manufactured by Sigma-Aldrich. A different siRNA against p21 (referred to as sip21 #1), containing a pool of siRNAs, was from Santa Cruz (Heidelberg). Control siRNA was obtained from Qiagen (Hilden). For irradiation, cells were exposed to a single dose of 2.0 Gy at room temperature by a linear accelerator (SL 75/5, Elekta, Crawley, UK) with 6 MEV photons/100 cm focus-surface distance and a dose rate of 4.0 Gy/min.

### Cellular extract preparation, Western blot analysis and indirect immunofluorescence

Cell lysis was performed using RIPA buffer (50 mM Tris pH 8.0, 150 mM NaCl, 1% NP-40, 0.5% Na-desoxycholate, 0.1% SDS, 1 mM NaF, 1 mM DTT, phosphatase and protease inhibitor cocktail tablets (Roche, Mannheim)). Western blot analysis was performed as previously described [55]. Cytoplasmic and nuclear fractionation was performed as instructed (Active Motif, La Hulpe, Belgium). Briefly, cells were harvested in ice-cold PBS with phosphatase inhibitors and resuspended in hypotonic buffer to swell the cell membranes. The cytoplasmic fractions were collected after adding detergent. The remaining pellet, the nuclei, was solubilized in a low-salt digestion buffer completed with protease inhibitor and PMSF followed by DNA digestion. Mouse monoclonal antibody against calnexin was from BD Biosciences (Heidelberg) and against lamin B1 from MBL (Woburn, USA). Mouse monoclonal antibodies against cyclin B1 and Plk1 were obtained from Santa Cruz. Rabbit monoclonal antibodies against caspase-3, phospho-Erk1/2 (Thr202/Tyr204) and p21, rabbit polyclonal antibodies against Akt, phospho-Akt (Ser473) and PARP were purchased from Cell Signaling. Rabbit polyclonal antibody against GAPDH was from abcam^®^ (Cambridge, UK). Mouse monoclonal anti-phospho-histone γ-H2AX (Ser139), rabbit polyclonal anti-Erk1/2 and anti-phospho-histone H3 (Ser10) were obtained from Merck Millipore. Mouse monoclonal antibody against β-actin was from Sigma-Aldrich. For indirect immunofluorescence staining cells were seeded on Nunc^TM^ Lab-Tek^TM^ II CC2^TM^ chamber slides from Thermo Fisher Scientific (Schwerte). Briefly, control or treated cells were fixed for 15 min with 4% PFA and permeabilized for 5 min with 0.2% Triton^TM^ X-100 at room temperature. The following primary antibodies were used for staining: polyclonal rabbit antibodies against pericentrin (abcam^®^) and 53BP1 (Novus, Cambridge, UK), monoclonal mouse antibodies against FITC-conjugated α-tubulin (Sigma-Aldrich), anti-phospho-histone γ-H2AX (Ser139) and human immune serum against centromere (anti-centromere antibody, ACA, ImmunoVision, Springdale, USA). FITC, Cy3 and Cy5-conjugated secondary antibodies were obtained from Jackson Immunoresearch (Newmarket, UK). DNA was stained using DAPI (4′,6-diamidino-2-phenylindole-dihydrochloride, Roche). Slides were examined using an AxioObserver.Z1 microscope (Zeiss, Göttingen) and images were taken using an AxioCam MRm camera (Zeiss). The immunofluorescence stained slides were further examined by confocal laser scanning microscope (CLSM, Leica CTR 6500, Heidelberg).

### Cell proliferation, cell cycle analysis and apoptosis assays

Cell proliferation assays were carried out by using Cell Titer-Blue^®^ Cell Viability Assay on treated cells in 96-well plates (Promega, Mannheim). 20 μl of CellTiter-Blue^®^ reagent was added to each well and then incubated at 37°C with 5% CO_2_ for 3 h before fluorescence reading using a Victor 1420 Multilabel Counter (Wallac, Finland). Cell cycle was analyzed using a FACSCalibur^TM^ (BD Biosciences), as described [55]. Briefly, cells were harvested, washed with PBS, fixed in chilled 70% ethanol at 4°C for 30 min, treated with 1 mg/ml of RNase A (Sigma-Aldrich) and stained with 100 μg/ml of propidium iodide (PI) for 30 min at 37°C. DNA content was determined. Early apoptosis (Ann+) and late apoptosis (Ann+PI+) were assessed using Vybrant™ apoptosis assay kit #2 according to the instructions (Molecular Probes, Leiden). Apoptosis was measured with a FACSCalibur^TM^ (BD Biosciences). The data were analyzed by using the cell cycle analysis software CellQuest (BD Biosciences). The activity of caspase-3/7 was measured in triplicate with Caspase-Glo^®^ 3/7 Assay as instructed (Promega).

### RNA extraction, real-time PCR and data analysis

Total RNAs were extracted with RNeasy kits according to the manual instructions (QIAGEN). Reverse transcription was performed using High-Capacity cDNA Reverse Transcription Kit as instructed (Applied Biosystems, Darmstadt). The TaqMan^®^ probes for p21 and GAPDH were obtained from Applied Biosystems. Real-time PCR was performed in triplicate with a StepOnePlus Real-time PCR System (Applied Biosystems) and the data were analyzed as described using the comparative ΔCT-method [56]. The results were represented as relative quantification (RQ) with minimum and maximum range. The RQ value for the DMSO control of HCT116 p21+/+ cells leads to the value RQ = 1.

### Senescence-associated β-galactosidase (SA-β-gal) assay and Ki-67 staining

Senescence-associated β-galactosidase (SA-β-gal) assay was performed at pH 5.9 as instructed [28]. Briefly, HCT116 p21+/+ and HCT116 p21−/− cells were seeded in chamber slides (Nunc^TM^ Lab-Tek^TM^ II CC2^TM^), treated for 96 h and stained with X-gal staining solution overnight at 37°C without CO_2_. The next day, cells were washed, fixed (5 min 4% PFA and 5 min with 0.2% Triton^TM^ X-100) and stained for immunohistochemical analysis with the monoclonal mouse anti-human Ki-67 antigen (Clone MIB-1, DAKO, Hamburg) by using the ImmunoCruz^TM^ mouse ABC staining system from Santa Cruz. The antibody complexes were visualized through exposure to 3-amino-9-ethylcarbazole (AEC) substrate (DAKO), as previously described [56]. The cells were observed with an AxioObserver.Z1 microscope (Zeiss), imaged with an AxioCam MRc camera (Zeiss) and the β-galactosidase positive cells were evaluated.

### Statistical analysis

Student's *t*-test (two tailed and paired or homoscedastic) was used to evaluate the significance of difference between HCT116 p21+/+ and HCT116 p21−/− or between U2OS/MDA-MB-231 cells treated with control siRNA or siRNA targeting p21. Difference was considered as statistically significant when p < 0.05.

## SUPPLEMENTARY MATERIAL FIGURES



## Abbreviations

Plk1: Polo-like kinase 1
PBD: Polo-box domain
PARP: poly (ADP-ribose) polymerase

## References

[1] Kreis NN, Louwen F, Yuan J (2014). Less understood issues: p21 in mitosis and its therapeutic potential. Oncogene.

[2] el-Deiry WS, Tokino T, Velculescu VE, Levy DB, Parsons R, Trent JM, Lin D, Mercer WE, Kinzler KW, Vogelstein B (1993). WAF1, a potential mediator of p53 tumor suppression. Cell.

[3] Gartel AL, Tyner AL (1999). Transcriptional regulation of the p21((WAF1/CIP1)) gene. Exp Cell Res.

[4] Cazzalini O, Scovassi AI, Savio M, Stivala LA, Prosperi E (2010). Multiple roles of the cell cycle inhibitor p21(CDKN1A) in the DNA damage response. Mutat Res.

[5] Gartel AL (2009). p21(WAF1/CIP1) and cancer: a shifting paradigm?. Biofactors.

[6] Warfel NA, el-Deiry WS (2013). p21WAF1 and tumourigenesis: 20 years after. Curr Opin Oncol.

[7] Kreis NN, Sanhaji M, Rieger MA, Louwen F, Yuan J (2013). p21Waf1/Cip1 deficiency causes multiple mitotic defects in tumor cells. Oncogene.

[8] de CG, Manning G, Malumbres M (2011). From Plk1 to Plk5: functional evolution of polo-like kinases. Cell Cycle.

[9] Strebhardt K (2010). Multifaceted polo-like kinases: drug targets and antitargets for cancer therapy. Nat Rev Drug Discov.

[10] Strebhardt K, Ullrich A (2006). Targeting polo-like kinase 1 for cancer therapy. Nat Rev Cancer.

[11] Louwen F, Yuan J (2013). Battle of the eternal rivals: restoring functional p53 and inhibiting Polo-like kinase 1 as cancer therapy. Oncotarget.

[12] Lenart P, Petronczki M, Steegmaier M, Di FB, Lipp JJ, Hoffmann M, Rettig WJ, Kraut N, Peters JM (2007). The small-molecule inhibitor BI 2536 reveals novel insights into mitotic roles of polo-like kinase 1. Curr Biol.

[13] Steegmaier M, Hoffmann M, Baum A, Lenart P, Petronczki M, Krssak M, Gurtler U, Garin-Chesa P, Lieb S, Quant J, Grauert M, Adolf GR, Kraut N, Peters JM, Rettig WJ (2007). BI 2536, a potent and selective inhibitor of polo-like kinase 1, inhibits tumor growth *in vivo*. Curr Biol.

[14] Rudolph D, Steegmaier M, Hoffmann M, Grauert M, Baum A, Quant J, Haslinger C, Garin-Chesa P, Adolf GR (2009). BI 6727, a Polo-like kinase inhibitor with improved pharmacokinetic profile and broad antitumor activity. Clin Cancer Res.

[15] Schoffski P, Awada A, Dumez H, Gil T, Bartholomeus S, Wolter P, Taton M, Fritsch H, Glomb P, Munzert G (2012). A phase I, dose-escalation study of the novel Polo-like kinase inhibitor volasertib (BI 6727) in patients with advanced solid tumours. Eur J Cancer.

[16] Keppner S, Proschak E, Kaufmann M, Strebhardt K, Schneider G, Spankuch B (2010). Biological impact of freezing Plk1 in its inactive conformation in cancer cells. Cell Cycle.

[17] Keppner S, Proschak E, Schneider G, Spankuch B (2011). Fate of primary cells at the G(1)/S boundary after polo-like kinase 1 inhibition by SBE13. Cell Cycle.

[18] Reindl W, Yuan J, Kramer A, Strebhardt K, Berg T (2008). Inhibition of polo-like kinase 1 by blocking polo-box domain-dependent protein-protein interactions. Chem Biol.

[19] Reindl W, Yuan J, Kramer A, Strebhardt K, Berg T (2009). A pan-specific inhibitor of the polo-box domains of polo-like kinases arrests cancer cells in mitosis. Chembiochem.

[20] Yuan J, Sanhaji M, Kramer A, Reindl W, Hofmann M, Kreis NN, Zimmer B, Berg T, Strebhardt K (2011). Polo-box domain inhibitor poloxin activates the spindle assembly checkpoint and inhibits tumor growth *in vivo*. Am J Pathol.

[21] Sanhaji M, Kreis NN, Zimmer B, Berg T, Louwen F, Yuan J (2012). p53 is not directly relevant to the response of Polo-like kinase 1 inhibitors. Cell Cycle.

[22] Waldman T, Kinzler KW, Vogelstein B (1995). p21 is necessary for the p53-mediated G1 arrest in human cancer cells. Cancer Res.

[23] Lera RF, Burkard ME (2012). High mitotic activity of Polo-like kinase 1 is required for chromosome segregation and genomic integrity in human epithelial cells. J Biol Chem.

[24] Sanhaji M, Louwen F, Zimmer B, Kreis NN, Roth S, Yuan J (2013). Polo-like kinase 1 inhibitors, mitotic stress and the tumor suppressor p53. Cell Cycle.

[25] McDonald ER, Wu GS, Waldman T, el-Deiry WS (1996). Repair Defect in p21 WAF1/CIP1 −/− human cancer cells. Cancer Res.

[26] Solier S, Pommier Y (2014). The nuclear gamma-H2AX apoptotic ring: implications for cancers and autoimmune diseases. Cell Mol Life Sci.

[27] Yim H, Erikson RL (2009). Polo-like kinase 1 depletion induces DNA damage in early S prior to caspase activation. Mol Cell Biol.

[28] Itahana K, Campisi J, Dimri GP (2007). Methods to detect biomarkers of cellular senescence: the senescence-associated beta-galactosidase assay. Methods Mol Biol.

[29] Gartel AL, Tyner AL (2002). The role of the cyclin-dependent kinase inhibitor p21 in apoptosis. Mol Cancer Ther.

[30] Roninson IB (2002). Oncogenic functions of tumour suppressor p21(Waf1/Cip1/Sdi1): association with cell senescence and tumour-promoting activities of stromal fibroblasts. Cancer Lett.

[31] Han Z, Wei W, Dunaway S, Darnowski JW, Calabresi P, Sedivy J, Hendrickson EA, Balan KV, Pantazis P, Wyche JH (2002). Role of p21 in apoptosis and senescence of human colon cancer cells treated with camptothecin. J Biol Chem.

[32] Suzuki H, Yabuta N, Okada N, Torigata K, Aylon Y, Oren M, Nojima H (2013). Lats2 phosphorylates p21/CDKN1A after UV irradiation and regulates apoptosis. J Cell Sci.

[33] Martinez LA, Yang J, Vazquez ES, Rodriguez-Vargas MC, Olive M, Hsieh JT, Logothetis CJ, Navone NM (2002). p21 modulates threshold of apoptosis induced by DNA-damage and growth factor withdrawal in prostate cancer cells. Carcinogenesis.

[34] Stivala LA, Cazzalini O, Prosperi E (2012). The cyclin-dependent kinase inhibitor p21CDKN1A as a target of anti-cancer drugs. Curr Cancer Drug Targets.

[35] Bunz F, Dutriaux A, Lengauer C, Waldman T, Zhou S, Brown JP, Sedivy JM, Kinzler KW, Vogelstein B (1998). Requirement for p53 and p21 to sustain G2 arrest after DNA damage. Science.

[36] Dulic V, Stein GH, Far DF, Reed SI (1998). Nuclear accumulation of p21Cip1 at the onset of mitosis: a role at the G2/M-phase transition. Mol Cell Biol.

[37] Stivala LA, Riva F, Cazzalini O, Savio M, Prosperi E (2001). p21(waf1/cip1)-null human fibroblasts are deficient in nucleotide excision repair downstream the recruitment of PCNA to DNA repair sites. Oncogene.

[38] Mauro M, Rego MA, Boisvert RA, Esashi F, Cavallo F, Jasin M, Howlett NG (2012). p21 promotes error-free replication-coupled DNA double-strand break repair. Nucleic Acids Res.

[39] Zhang Y, Fujita N, Tsuruo T (1999). Caspase-mediated cleavage of p21Waf1/Cip1 converts cancer cells from growth arrest to undergoing apoptosis. Oncogene.

[40] van Vugt MA, Bras A, Medema RH (2005). Restarting the cell cycle when the checkpoint comes to a halt. Cancer Res.

[41] Abbas T, Dutta A (2009). p21 in cancer: intricate networks and multiple activities. Nat Rev Cancer.

[42] de RC, Depamphilis ML, Ullah Z (2014). Cytoplasmic Localization of p21 Protects Trophoblast Giant Cells from DNA Damage Induced Apoptosis. PLoS One.

[43] Li Y, Dowbenko D, Lasky LA (2002). AKT/PKB phosphorylation of p21Cip/WAF1 enhances protein stability of p21Cip/WAF1 and promotes cell survival. J Biol Chem.

[44] Zhou BP, Liao Y, Xia W, Spohn B, Lee MH, Hung MC (2001). Cytoplasmic localization of p21Cip1/WAF1 by Akt-induced phosphorylation in HER-2/neu-overexpressing cells. Nat Cell Biol.

[45] Vincent AJ, Ren S, Harris LG, Devine DJ, Samant RS, Fodstad O, Shevde LA (2012). Cytoplasmic translocation of p21 mediates NUPR1-induced chemoresistance: NUPR1 and p21 in chemoresistance. FEBS Lett.

[46] Heliez C, Baricault L, Barboule N, Valette A (2003). Paclitaxel increases p21 synthesis and accumulation of its AKT-phosphorylated form in the cytoplasm of cancer cells. Oncogene.

[47] Cheng X, Xia W, Yang JY, Hsu JL, Chou CK, Sun HL, Wyszomierski SL, Mills GB, Muller WJ, Yu D, Hung MC (2010). Activation of p21(CIP1/WAF1) in mammary epithelium accelerates mammary tumorigenesis and promotes lung metastasis. Biochem Biophys Res Commun.

[48] Yim H (2013). Current clinical trials with polo-like kinase 1 inhibitors in solid tumors. Anticancer Drugs.

[49] Munoz-Espin D, Canamero M, Maraver A, Gomez-Lopez G, Contreras J, Murillo-Cuesta S, Rodriguez-Baeza A, Varela-Nieto I, Ruberte J, Collado M, Serrano M (2013). Programmed cell senescence during mammalian embryonic development. Cell.

[50] Storer M, Mas A, Robert-Moreno A, Pecoraro M, Ortells MC, Di G V, Yosef R, Pilpel N, Krizhanovsky V, Sharpe J, Keyes WM (2013). Senescence is a developmental mechanism that contributes to embryonic growth and patterning. Cell.

[51] Kim HJ, Cho JH, Kim JR (2013). Downregulation of Polo-like kinase 1 induces cellular senescence in human primary cells through a p53-dependent pathway. J Gerontol A Biol Sci Med Sci.

[52] Campisi J (2013). Aging, cellular senescence, and cancer. Annu Rev Physiol.

[53] Gordon RR, Nelson PS (2012). Cellular senescence and cancer chemotherapy resistance. Drug Resist Updat.

[54] Cagnol S, Chambard JC (2010). ERK and cell death: mechanisms of ERK-induced cell death--apoptosis, autophagy and senescence. FEBS J.

[55] Kreis NN, Sommer K, Sanhaji M, Kramer A, Matthess Y, Kaufmann M, Strebhardt K, Yuan J (2009). Long-term downregulation of Polo-like kinase 1 increases the cyclin-dependent kinase inhibitor p21(WAF1/CIP1). Cell Cycle.

[56] Muschol-Steinmetz C, Friemel A, Kreis NN, Reinhard J, Yuan J, Louwen F (2013). Function of survivin in trophoblastic cells of the placenta. PLoS One.

