# Supplementary material for: Loss of p21Cip1/CDKN1A renders cancer cells susceptible to Polo-like kinase 1 inhibition
Source: Oncotarget. 2014 Dec 2;6(9):6611–26. doi: 10.18632/oncotarget.2844 (PMC4466638; doi:10.18632/oncotarget.2844)
Supplement: Supplementary file 1 [file oncotarget-06-6611-s001.pdf]

Loss of p21$^{Cip1/CDKN1A}$ renders cancer cells susceptible to Polo-like kinase 1 inhibition

Supplementary Material

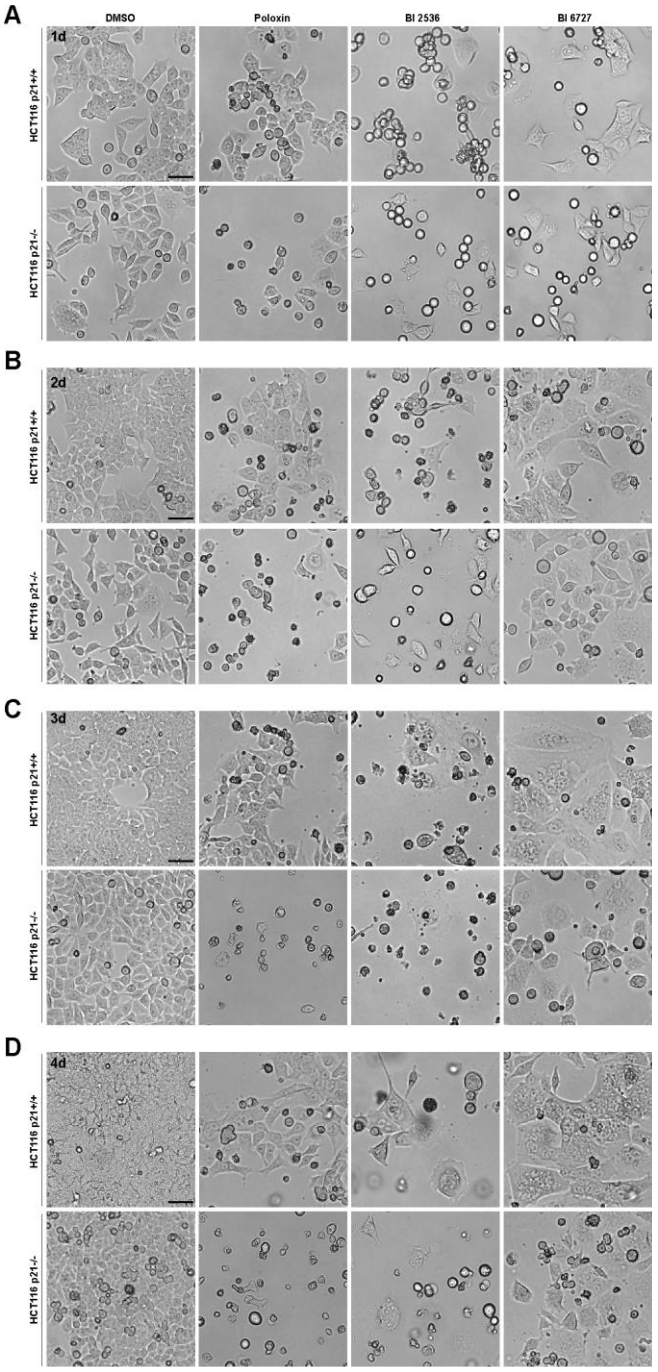

Figure S1: Images of bright-field microscopy upon Plk1 inhibitor treatment. HCT116 p21+/+ cells or HCT116 p21-/- cells were treated with 25 µM Poloxin, 25 nM BI 2536 or 25 nM BI 6727 for 1 day (A), 2 days (B), 3 days (C) and 4 days (D), respectively. Bright-field images were taken. Scale bar: 50 µm.

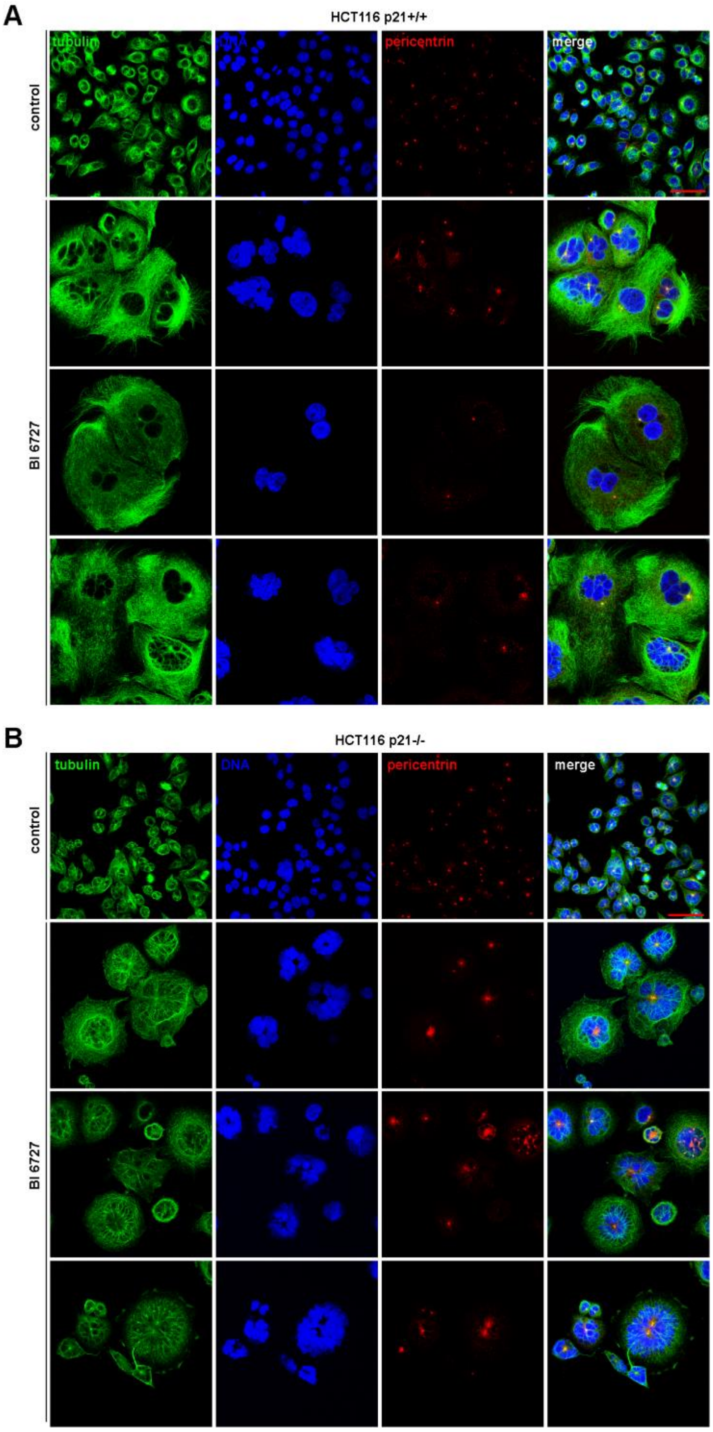

Figure S2: Immunofluorescence staining of long-term treated cells. HCT116 p21+/+ cells (A) and HCT116 p21-/- cells (B) were untreated or treated with 25 nM BI 6727 for 96 h. Cells were stained with indicated antibodies and images were taken by confocal laser scanning microscopy. Scale bar: 50 µm.
